# Factors impacting the evidence-based assessment, diagnosis and management of Acute Charcot Neuroarthropathy: a systematic review

**DOI:** 10.1186/s13047-021-00469-5

**Published:** 2021-04-07

**Authors:** D. Diacogiorgis, B. M. Perrin, M. I. C. Kingsley

**Affiliations:** 1 Department of Podiatry and Allied Health Assistants, Ballarat Health Services, Ballarat, Australia; 2 La Trobe Rural Health School, College of Science, Health and Engineering, La Trobe University, Bendigo, Australia; 3 Holsworth Research Initiative, La Trobe Rural Health School, College of Science, Health and Engineering, La Trobe University, Bendigo, Australia; 4 Department of Exercise Sciences, Faculty of Science, University of Auckland, Auckland, New Zealand

**Keywords:** Diabetes, Charcot, Foot, Peripheral neuropathy, Neuroarthropathy

## Abstract

**Background:**

Acute Charcot Neuroarthropathy (CN) is a destructive condition that is characterised by acute fractures, dislocations and joint destruction in the weight-bearing foot. The acute phase is often misdiagnosed and can rapidly lead to devastating health outcomes. Early diagnosis and management of CN is imperative to attenuate progression of this condition. Consequently, timely evidence-based assessment, diagnosis and management of acute CN is imperative.

**Objective:**

To identify the factors that impact the delivery of evidence-based care in assessment, diagnosis and management of people with acute CN.

**Method:**

Systematic searches were conducted in four databases to identify studies in English that included factors that impact the delivery of evidence-based care in the assessment, diagnosis and management of people with acute CN. Articles and consensus/guideline documents were assessed for inclusion by the researchers and disagreements were resolved through consensus. Additionally backward citation searching was used to source other potentially relevant documents. Information relevant to the research question was extracted and thematic analyses were performed using qualitative synthesis.

**Results:**

Thirty-two articles and four additional consensus/guideline documents were included for data extraction and analyses. Information related to the research question was of expert opinion using the National Health and Medical Research Council (NHMRC) Levels of Evidence guidelines. Themes explaining practices that deviated from evidence-based care in assessment, diagnosis and management of acute CN centred around patient, health professional and health organisation/environmental. Delay to diagnosis is particularly influenced by the patient’s knowledge of when to seek help, practitioner knowledge in knowing how to recognise and refer for appropriate immediate care, confusion in imaging and offloading and geographical and local health service resources to appropriately manage the condition.

**Conclusion:**

Individual and health professional awareness and geographical barriers are key challenges to the effective delivery of evidence-based assessment, diagnosis and management of people with acute CN. Acute CN represents a medical emergency warranting the need for expedited assessment, diagnosis and management by appropriately trained health professionals in the appropriate.

## Introduction

Acute Charcot Neuroarthropathy (CN) is an end stage complication in people with a diagnosis of diabetes or other less common conditions that cause peripheral neuropathy [1]. It is a progressive complication of the neuropathic foot initially characterised by gross inflammation of the foot or ankle, redness, heat and ultimately bony destruction if left untreated [2]. What triggers the gross inflammatory process is not well understood, with research into gene expression and early detection methods for CN proving inconclusive [1]. The clinical manifestations such as swelling, redness and changed structure of the foot in CN are more clearly recognised by trained health professionals once they occur and are clearly articulated in clinical guidelines to aid clinical decision-making [3]. However, broader awareness amongst health professionals and patients and carers themselves is limited, which can lead to delayed diagnosis and treatment of acute CN [1]. If left untreated, or treatment is delayed, acute CN can lead to devastating health complications such as foot ulceration and lower limb amputation [4].

An appropriate multidisciplinary model of care has been shown to improve diabetes-related foot complications, including CN [4, 5]. If appropriately managed, acute CN can completely resolve within 12–14 months [6]. The accepted standard for the management of acute CN is clear and recommends the application of offloading in order to reduce discomfort, inflammation and potential change to the bony structure of the foot [1, 4, 6]. The gold standard of direct treatment is total contact casting (TCC), which provides irremovable offloading in an effort to protect the structure of the foot [1, 4, 6]. Less optimal options include removable devices such as Charcot Restraint Orthotic Walkers (CROW), that are custom made or Controlled Ankle Motion (CAM) walkers, which are not custom made. Regular medical and surgical monitoring to guide assessment and treatment planning is an important adjunct to the treatment process [1].

The current evidence base relating to acute CN focuses on the biomedical aspects of the assessment, diagnosis and management of this condition [1]. Evidence-based pathways, guidelines and consensus documents for the management of acute CN exist, but are more applicable for large tertiary-level health service providers [1, 3, 4, 6] and can be challenging to translate into practice [4]. Guidelines are geared towards tertiary hospitals that have access to multiple disciplines and specialties, particularly in metropolitan areas, that enable them to be easily implemented [4]. The complex nature of CN and the multidisciplinary needs of the patient means that there can be challenges in implementing best practice health care. For example, patients with acute CN or other diabetes-related foot problems often access health services through a variety of different entry points such as primary care, hospital outpatient clinics and emergency departments [7], which can delay diagnosis and implementation of appropriate treatment [8, 9]. Regional and rural health services also face further challenges, as fewer knowledgeable health professionals are often spread across a larger geographic area, and there are higher levels of social disadvantage [10]. In Australia, podiatry services are often the principle contact for management of acute CN in consultation with specialists, general practitioners and the broader multidisciplinary team [3, 4]. Knowledgeable clinicians such as skilled podiatrists are mostly located within metropolitan or large regional centres [4].

It is clear that acute CN is a complex devastating condition for those who are diagnosed with it, which inherently poses a challenge to health professionals to manage it in a way consistent with the evidence. Therefore, the aim of this systematic review was to determine the factors that influence the evidence-based assessment, diagnosis and management of acute CN.

## Method

### Search strategy

Four literature databases: Medline (OvidSP), Pubmed (NCBI), Embase (OvidSP) and The Cumulative Index for Nursing and Allied Health Literature [CINAHL] (EBSCO) were searched from inception to 9th May 2020.

The search strategy included three constructs: ‘Population’, ‘Core Concept’ and ‘Context’ [11]. Population included “Charcot” and not “Charcot Marie Tooth”. Core Concept search terms were selected to describe factors impacting the evidence-based care in acute CN, and were identified from titles of articles in the reference list of a systematic review that lead to the development of a treatment pathway for CN [6]. Multiple combinations of terms were tested to optimise the combination of search terms. Search terms in the Context construct were “assessment”, “diagnosis” and “management”. Core Concept and Context terms were combined with ‘OR’. Finally, the constructs were combined with the ‘AND’ operator. An example of the Pubmed search string is provided in Table 1.

**Table 1:** Pubmed database search string

Database	Search String	Search	Outcome
PubMed	((((Charcot) NOT Charcot Marie Tooth)) AND (((((((((Cost) OR Comparison) OR Study) OR Audit) OR Quality of Life) or Outcome) or Experience) OR Knowledge)) AND (((Assessment) OR Diagnosis) OR Management)	English language, Title	314

As information was anticipated to come from a broad scope of literature, including both qualitative and quantitative research, reference lists from included articles were searched (backward citation searching) for other documents, such as guidelines and/or consensus documents, to identify potentially relevant documents.

### Eligibility criteria

This systematic review included literature that focuses on people diagnosed with acute CN in the context of underlying conditions that cause peripheral neuropathy (e.g. diabetes, progressive neurological disorders). The literature that was included identified factors that impact the delivery of evidence-based care in the assessment, diagnosis and management of people with acute CN. The exclusion criteria were: (1) non-English language; (2) not acute CN; (3) does not consider factors that impact the delivery of evidence-based care in CN; and (4) does not consider assessment, diagnosis or management.

### Review process, data extraction and themes of interest

Following the removal of duplicates, two reviewers independently assessed articles for inclusion by abstract and then full-text in a two stage process. Where both reviewers did not agree to include or exclude articles, disagreements were resolved by consensus with a third reviewer. To aid critical appraisal, the level of evidence for findings that addressed the study question was assessed using the National Health and Medical Research Council Levels of Evidence (NHMRC) [12]. This was required because much of the included data were found from analysis of secondary variables or presented as discussion points. For example, if a study had a directly relevant research question and utilised meta-analyses it was rated as level I evidence; or if the information was based on discussion by author(s) only relating to secondary considerations, the evidence was rated according to the information relevant to the research question (e.g. expert opinion [EO]).

## Results

**Fig. 1:**
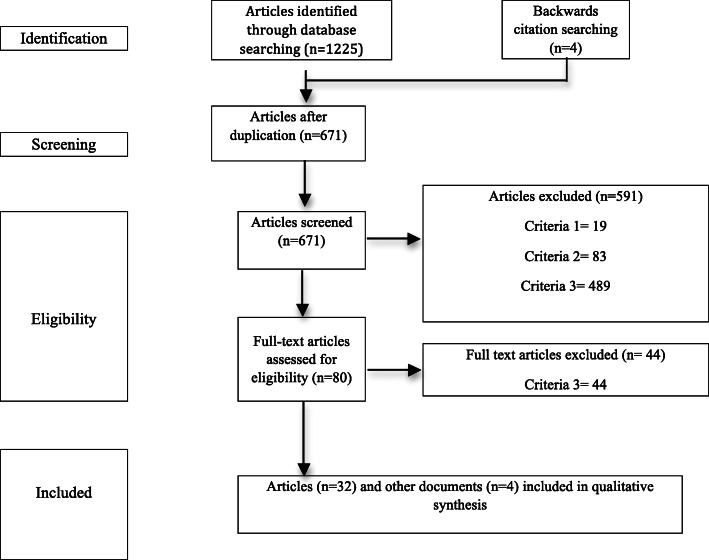
PRISMA flow diagram of the search, record exclusions and included studies for qualitative synthesis [13]

The initial search identified 667 unique articles. After application of the exclusion criteria, 32 articles and four other documents (guidelines and consensus documents) were included in this systematic review (Fig. 1).

Table 2 shows the characteristics of the included documents and a description of the relevant factors that influence the evidence-based assessment, diagnosis and/or management of acute CN. As identified in Table 2, extracted evidence was of low quality and sourced mainly from expert opinion, reviews, consensus positions, case series and case studies. Only one article directly addressed the current research question [7]. In most of the articles, discussion of the specific factors that impacted on the delivery of evidence-based care were from secondary observations or simply opinion by authors in the discussion sections, often when describing limitations of the studies. Although there were limited studies that directly answered the research question, the information extracted from the included articles was consolidated into broad themes in order to contextualise the challenges in implementing evidenced-based practice for acute CN. These themes were health organisational and environmental factors, individual factors and health professional factors (Table 2).

**Table 2:** Summary of included articles and other documents: level of evidence, relevant themes and information contained within each article that addressed the research question under the contexts of assessment, diagnosis and management

Author(s)	Study Design/Country	Level of Evidence	Themes	Assessment	Diagnosis	Management
Blume et al., 2014 [14].	Literature review/United States of America	EO	Health Organisation Health Professional	Health professional knowledge to recognise symptoms of CN	Health professional knowledge to utilise the appropriate pathology and imaging to diagnose CN. Resource limitation leads to more use of x-ray	Health professional knowledge to utilise and apply the appropriate form of offloading
Bullen et al,. 2018 [15].	Delphi/ Scotland	EO	Individual Health Professional	Health professional knowledge to appropriately prepare individuals for the potential onset of CN	Nil	Health professional capacity to educate the patient to understand importance of offloading. Literacy capacity of individual
Chantelau, 2005 [16].	Case Controlled study/ Germany	III-2	Health Professional	Health professional knowledge to recognise symptoms of CN	Delayed diagnosis. Health professional knowledge and confusion as to the appropriate form of imaging to use, Knowledge limitation leads to more use of x-ray	Delayed diagnosis leads to delayed treatment such as offloading
Chantelau et al., 2007 [17].	Case Series/ Germany	IV	Individual Health professional	Early symptoms such as deep dull aches often unrecognised by patient leading to delayed presentation. Health professional knowledge to recognise symptoms of CN	Health professional knowledge and confusion as to the appropriate form of imaging to use	Delayed diagnosis leads to delayed treatment. Health professional knowledge of utilizing the appropriate form of offloading impacts treatment duration
Chantelau et al., 2013 [18].	Retrospective Cohort study/ Germany	IV	Individual Health Professional	Early symptoms such as deep dull aches often unrecognised by patient	Delayed diagnosis. Health professional knowledge and confusion as to the appropriate form of imaging to use. Knowledge limitation leads to more use of x-ray	Delayed diagnosis leads to delayed treatment. Health professional knowledge as to when to transition patient between various forms of offloading.
DiDomenico et al., 2018 [19].	Literature review/ United States of America	EO	Individual Health Professional	Underlying comorbidities of the individual patient such as diabetes and obesity has an impact on implementation of best practice	Nil	Complex condition requiring complete lifestyle modification
Dixon et al., 2017 [20].	Retrospective case series/ New Zealand	IV	Individual Health Professional	Early symptoms such as deep dull aches often unrecognised by patient leading to delayed presentation (17 weeks). Health professional knowledge (GP) to recognise symptoms of CN and refer appropriate service	Delayed diagnosis. Health professional knowledge and confusion as to the appropriate form of imaging to use. Knowledge limitation leads to more under utilization of MRI	Health professional knowledge of when to transition patients to footwear
Farid et al., 2008 [2].	Case Study/ United States of America	IV	Individual Health Professional	Underlying comorbidities of the individual patient such as diabetes and obesity has an impact on implementation of best practice	Nil	Health professional limited experience in appropriately being able to apply TCCs. Health professional ability to properly explain the treatment regimen to individual. Individual compliance with lack of understanding of the complexities of treatment of CN
Frykberg et al., 2012 [5].	Round Table Discussion/ United States of America	EO	Individual Health Professional	Underlying comorbidities of the individual patient such as diabetes, obesity has an impact on implementation of best practice	Nil	Health professional confusion as to which surgical procedure to use. Concern regarding informed consent, litigation and compliance with treatment protocols
Gil et al., 2013 [21].	Case Controlled study/ United States of America	III-2	Individual	Nil	Nil	Health professional confusion as to which surgical procedure to use. Concern regarding informed consent, litigation and compliance with treatment protocols
Gooday et al., 2020 [22].	Systematic Review/ United Kingdom	EO	Health professional	Nil	Nil	Health professional monitoring techniques inconsistent.
Jansen et al., 2016 [23].	Qualitative, Survey/ Denmark	IV	Health Professional	Health professional knowledge to recognise symptoms of CN	Health professional knowledge and confusion as to the appropriate form of imaging to use. Knowledge limitation leads to more use of x-ray	Health professional monitoring techniques inconsistent. Health professional limited experience in appropriately being able to apply TCCs
Jeffcoate, 2015 [24].	Literature review/ United Kingdom	EO	Individual Health Professional	Early symptoms such as deep dull aches often unrecognised by patient leading to delayed presentation. Health professional knowledge to recognise symptoms of CN	Health professional knowledge and confusion as to the appropriate form of imaging to use. Knowledge limitation leads to more use of x-ray	Health professional limited experience in appropriately being able to apply TCCs. Inconsistent treatment protocols and lack of agreed outcome measures
Loupa et al., 2019 [25].	Case study/ Greece	IV	Individual Health Professional	Health professional misdiagnosis and delayed diagnosis, lack of awareness	Health professional knowledge and confusion as to the appropriate form of imaging to use. Knowledge limitation leads to more use of x-ray	Delayed treatment as a result of delayed diagnosis. Individual compliance through treatment process impacted success
McIntyre et al., 2007 [26].	Retrospective audit - case control study. Qualitative structured interviews/ Canada	IV	Environment Individual	Cultural environment and proximity to services. Aboriginal patients younger, less education, employment, greater burden of disease, financial disadvantage, less patient understanding of their condition	Nil	Nil
Metcalf et al., 2018 [27].	Retrospective audit - case control study/ United Kingdom	IV	Health Professional	Health professional knowledge to recognise symptoms of CN and refer appropriate service	Nil	Nil
Milne et al., 2013 [6].	Systematic review/ Australia	EO	Health Organisation Environment Individual Health Professional	Health professionals require a high index of clinical suspicion otherwise mis/delayed diagnosis occurs. Critical gap in education of the community and health professional knowledge of CN and prompt referral to a multidisciplinary clinic	Health professional knowledge to utilise the appropriate pathology and imaging to diagnose CN. Resource limitation leads to more use of x-ray	Management driven by expert consensus rather than rigorous evidence-based practice. Health professional expertise in the application of TCC critical and resource intensive. Variability in the advice provided by health professionals regarding protected weightbearing. Adherence of patients to the use of removable cast walkers variable. Geographical location is a consideration in the treatment of CN
O'Loughlin et al., 2017 [28].	Retrospective audit - case series/ Ireland	III-2	Health Professional	Health professional knowledge of CN results in mis/delayed diagnosis. Patient presentation not timely and the urgent nature of condition not clear when they experience symptoms. There is an underrepresentation of CN in the community.	Health professional knowledge and confusion as to the appropriate form of imaging to use. Knowledge limitation leads to more use of x-ray	Health professional knowledge gap leads to delayed treatment. More frequent ulceration in the context of acute CN with removable cast walkers than non-removable cast walkers. Outcomes better with non-removable cast walkers. Significant health burden once ulcer occurs
Pakarinen wt al., 2009 [29].	Cross Sectional study/ Finland	III-2	Individual	Nil	Diagnosis made within three months associated with better patient physical and social outcomes.	Social functioning and physical condition of the patient decreases with non-surgical treatment
Perrin et al., 2010 [30].	Case Study/ Australia	IV	Health Professional	Health professional misdiagnosis and delayed diagnosis, lack of awareness	Health professional knowledge and confusion as to the appropriate form of imaging to use. Knowledge limitation leads to more use of x-ray	Delayed treatment as a result of delayed diagnosis Early implementation of offloading of TCC would have been more ideal
Petrova et al., 2017 [31].	Literature review/ United Kingdom	EO	UK/Kings College NHS Trust Foundation	Health professionals high index of suspicion necessary.	Nil	Health professional limited experience in appropriately being able to apply TCCs. Inconsistent treatment protocols
Rettedal et al., 2018 [32].	Retrospective audit - case series/ United States of America	IV	Individual Health Professional	Nil	Nil	Anatomic location of CN and patient medical factors such as glycated haemoglobin, nutrition can determine outcome of surgical reconstruction, patient psychosocial factors and family support
Robinson et al., 2015 [33].	Literature review and case review/ United States of America	EO	Individual	Nil	Nil	Patient education and clinician understanding of clinical parameters underpinning CN management imperative and could increase compliance
Sanders, 2008 [34].	Literature review/ United States of America	EO	Health professional	Health professional observation is paramount, high level of clinical suspicion necessary, recognition of acute CN is variable	Nil	Health professional confusion as to which surgical procedure to use. Concern regarding informed consent, litigation and compliance with treatment protocols
Schmidt et al., 2017 [7].	Survey/ United States of America	IV	Individual Health Professional	Health professional poor knowledge leads to misdiagnosis Early stages of the condition not recognised by the patient as they are neuropathic resulting in referral delay	Ambiguous diagnosis criteria means actual incidence and prevalence may not be known	Nil
Schmidt et al., 2018 [35]	Literature review/ United States of America	EO	Individual Health Professional	Health professionals must rely on clinical judgement. Health professional poor knowledge leads to misdiagnosis. Non-specific clinical findings. Patient unable to detect symptoms	Ambiguous diagnostic criteria	Nil
Schmidt et al., 2019 [36]	Observational Cohort study/ United States of America	III-2	Health Professional	Health professional poor knowledge leads to misdiagnosis	Nil	Primary outcomes improved with dedicated specialist care. Improved patient education and compliance improves outcomes.
Sinacore et al., 1999 [37].	Literature review/ United States of America	EO	Individual Health professional	Patients delay in seeking assessment/management, identification and appropriate referral by clinicians. Patient understanding of acute CN a risk factor	Nil	No clear indicators of when a patient can transition between the varies stages of restricted mobilisation, extent of injury and pattern, greater weightbearing mid foot and hindfoot-healing longer
Wade, 2016 [38].	Literature review/ United States of America	EO	Individual Health professional	Health professionals require a high index of clinical suspicion otherwise mis/delayed diagnosis occurs. Critical gap in education of the community and health professional knowledge of CN and prompt referral to a multidisciplinary clinic	Negative x-ray can delay healing, diagnosis not always confirmed by imaging	Patient education and clinician understanding of clinical parameters underpinning CN management imperative and could increase compliance
Welch et al., 2014 [39].	Survey/ United Kingdom	IV	Health Organisation Health professional	Health professional knowledge, lack of confidence, unwillingness to perform crucial foot assessments if clinical indicators not present, poor resource, lack of time, incomplete assessments	Health professional knowledge to utilise the appropriate pathology and imaging to diagnose CN. Resource limitation leads to more use of x-ray	Nil
Wennberg et al., 2017 [40].	Cross Sectional study/ Sweden	III-2	Health Organisation Individual Health professional	Lack of recognition, delayed assessment/diagnosis	Health professional knowledge to utilise the appropriate pathology and imaging to diagnose CN. Resource limitation leads to more use of x-ray	Limited treatment options, MRI would provide earlier diagnosis, anxiety and depression of patient
Wukich et al., 2009 [41].	Literature review/ United States of America	EO	Health Organisation Individual Health professional	Missed cases, high index of suspicion, clinician dependant, delayed patient presentation,	Health professional knowledge to utilise the appropriate pathology and imaging to diagnose CN. Resource limitation leads to more use of x-ray	Patient education and clinician understanding of clinical parameters underpinning CN management imperative and could increase compliance
Baker IDI, 2011 [4].	Guideline/ Australia	EO	Australia	Access to health services in rural remote areas	Nil	Nil
Diabetes Canada, 2008 [42].	Guideline/ Canada	EO	Canada/ Diabetes Canada	High degree of suspicion necessary	Nil	Nil
IWGDF, 2019 [3]	Guideline/ Netherlands	EO	Netherlands/Meeting of experts	High degree of suspicion necessary	Nil	Nil
Rogers et al., 2011 [1].	Expert Opinion/ France	EO	Paris/Meeting of experts	Early detection on inflammation. Health professional knowledge	Nil	Nil

The information extracted from the included articles was consolidated into broad themes in order to contextualise the challenges in implementing evidenced-based practice for acute CN. These themes were health organisational and environmental factors, individual factors and health professional factors

A fundamental factor impacting best practice that cut across all themes was a delay in timely assessment, diagnosis and management. This delay could be related to a patient’s lack of awareness of the condition resulting in delayed presentation to an appropriate service, lack of health professional knowledge of CN and applied management skills, lack of patient proximity to services and access, and health service protocol on the management of CN [1, 3–7, 14–38, 40–43].

### Health Organisational and environmental factors

Health organisations often do not have the expertise and capacity to assess, diagnose and manage acute CN in a timely and efficient manner because of a lack of skilled clinicians and equipment available to diagnose and then manage acute CN [14, 17, 39], and this is especially important in rural and remote areas [14, 17]. Schmidt et al. identified that reduced investment in health professional training, and lack of access to diagnostic imaging modalities such as Magnetic Resonance Imaging (MRI) can result in poorer health outcomes for the patient with CN [7]. Unclear pathways and treatment protocols can lead to confusion and mismanagement of CN [20].

Geographic location was identified by two included articles as impacting on evidence-based care [4, 26]. Access to specialist care in dispersed geographical rural and remote communities was identified as a barrier to care [26]. Further, this reduced proximity to services compounds the challenge faced by culturally disadvantaged communities with low education, low income and greater risk of complication to access services in a timely way [4, 26]. Increased distance to services has been identified as a factor influencing the appropriate management of diabetes foot complications including CN. This could be due to a lack of skilled health professionals in rural and regional areas and the prohibitive travel required to access the appropriate health care [4].

### Individual factors

Of the included documents, 12 articles identified patient knowledge of what to look for and when to seek help as a key barrier to the delivery of evidence-based health care in acute CN. A delay in diagnosis and treatment was often due to the patient’s inability to identify the imminent onset of the condition, which could be as a result of an inability to identity a precipitating traumatic episode, their disregard of painful symptoms (e.g. dull ache) and a general lack of awareness of their foot-health [7, 15, 17, 18, 20, 24, 35].

Underlying health issues were implicated in three articles specifically as affecting diagnosis and implementation of management strategies [5, 19, 24]. Diabetes and obesity resulting in hyperglycaemia leading to peripheral neuropathy diminishes the patient’s ability to detect an issue and seek urgent care [5, 19, 24]. Frykberg et al. [5] also highlighted poor mental health in this cohort of patients as compared to those with heart disease, with this alone increasing the difficulty with compliance of treatment protocols and gaining informed consent. Six articles referred to the importance of informed consent as a means of the patient to properly understand the risk associated with acute CN [5, 18, 21, 24, 33, 43]. Informed consent was identified as a factor with patients not comprehending the reasons for undertaking a particular treatment and therefore not being agreeable to the process. Adherence to treatment regimens for acute CN was also challenged by a decline in quality of life particularly in relation to changed body shape and restriction of activities of daily living or occupation due to the high demand associated with the use of offloading devices [21, 25, 29, 32, 36, 40, 41].

### Health professional factors

The successful management of CN is highly dependent on access to appropriately skilled health professionals and was identified by 26 of the included articles [23, 24, 31]. Physician or health professional knowledge of how to recognise and assess for acute CN and mistaking it for a number of differential diagnoses is commonly identified as a barrier to evidence-based care [7, 28, 35, 39–41]. Lacking awareness and a high index of suspicion was also identified as an underlying factor impacting physician action and referral to appropriate care [7, 27, 30, 34–36]. For example, if patients attend their general practitioner as their primary health carer the patient may not receive the appropriate clinical escalation and timely healthcare by appropriately skilled health professionals [14, 20, 23, 27]. A high index of suspicion of CN in knowledgeable physicians is important, and Rogers et al. [1], International Working Group on the Diabetic Foot [3], and Diabetes Canada [42] are consistent in recommending that treatment should commence prior to diagnosis when there is high degree of suspicion that CN is likely.

Confusion around what type of diagnostic tool is effective in diagnosis and monitoring was highlighted in nine articles, with the choice of imaging modality and thresholds for diagnosis influencing timely diagnosis, commencement of management and cessation of offloading [6, 7, 16, 18, 20, 28, 30, 38, 40]. Whether to utilise x-ray or MRI and the difficulty of differentiating between osteomyelitis and acute CN was regularly identified [16–18, 20, 24]. Reference to the use of serial x-ray as an appropriate means of diagnosis was overshadowed through comparison of MRI and x-ray suggesting that early episodes of acute CN can be missed with x-ray [6, 16, 18, 30, 31, 40], and MRI should be the first line imaging modality [1, 3]. However, x-rays were identified as a useful option [6, 16, 18, 30, 31, 40] and two guidance documents support the use of x-ray or MRI to diagnose and manage acute CN [1, 3]. Other forms of imaging such as positron emission tomography (PET), computed tomography (CT) and bone scintigraphy are noted as potential forms of imaging that could and should be used during the diagnostic and management phases of acute CN [14].

Inconsistencies are reported among health professionals about the choice of immediate offloading of the foot implemented for the management of CN [5, 6, 14, 16, 30, 41]. The irremovable TCC is seen as the gold standard for immediate offloading and more favourable than the use of removable modalities. However, the latter is often used partly due to physician inexperience and skill in the application of a TCC, and/or patient acceptance and lack of adherence to the gold standard treatment plan [25, 28, 36]. Unfortunately, physician knowledge of TCC and the specific indications, prescription, monitoring and removal was deemed to be highly variable [14, 17, 22, 37].

## Discussion

To the authors’ knowledge this systematic review is the first attempt to specifically identify the factors that can impact the delivery of evidence-based care in the assessment, diagnosis and management of people with acute CN. The paucity of high-level evidence in this area was clear. With the exception of Schmidt et al. [7], evidence was obtained from observations of secondary outcome variables in the studies or discussion by authors in the discussion sections, often when describing limitations of studies. The primary outcome from this study was the identification of the dearth of evidence to guide health professionals on how to implement timely assessment diagnosis and management of acute CN in their local health setting.

While the relevant global guidance and clinical pathway publications directly describe the best available evidence base for assessment, diagnosis and management of acute CN, little attention has been given to factors that may impact on the delivery of this care [1, 3, 4, 42]. This finding is not surprising because the biopsychosocial barriers influencing foot-health outcomes in people with diabetes and potentials barriers to translating evidence into practice is only recently gaining attention [44, 45]. This is particularly concerning as acute CN has been described as a medical emergency, where treatment can be very effective in mitigating pathophysiological deterioration and subsequent gross structural and functional damage to the foot [6].

The lack of high-quality, focussed research in this area is a problem for the foot-health of people with diabetes and particularly acute CN. Maintaining the foot-health of people with diabetes requires significant engagement of both the patient and skilled, knowledgeable, multiple health disciplines [1, 4]. This engagement with foot-health can add to an already high burden on a person managing their diabetes, and generally occurs in isolation to health professionals. Furthermore, a coordinated multidisciplinary health care approach is needed. Therefore, there are likely to be many factors that can impact on the delivery of evidence-based care and eventual health outcomes. This review has identified several factors across consistent themes that are likely to be important to consider in order to ensure delivery of evidenced-based care to people with acute CN. These themes incorporate the entire health-care spectrum of acute CN, involving the person with acute CN and the environment they live in and the health professionals and health organisations they interact with.

A lack of understanding of the condition, both from individuals with diabetes and health professionals, could be a major barrier to implementing evidenced based care for acute CN. An individual’s lack of understanding of acute CN and particularly the inability to identify acute CN is a consistent theme that evolved from this review and could be at the forefront of why individuals at risk do not present for care early enough [7, 16, 18, 32, 34 l, 36]. Even though acute CN is often associated with observable signs such as redness, swelling, heat [6] and sometimes pain, people often do not recognise this as acute CN and a delay in seeking medical assistance is often the result. This might be partially explained by the strong association of acute CN with peripheral neuropathy [5, 24], and it is possible that a misunderstanding of the general foot-health consequences of diabetes can contribute to patient uncertainty and inappropriate action in the event of acute foot-health complications [45, 46].

Acute CN is also poorly understood by health professionals. The one study that directly addressed the current research question found a high variability in the level of knowledge of assessment, diagnosis and management of acute CN by a cohort of physicians, which resulted in a significant delay in diagnosis of acute CN [7]. This was underpinned by an inability of health professionals to objectively identify the signs and symptoms of acute CN. A general lack of health professional understanding across health disciplines in various settings has been implicated in the variation of care for people with acute CN and the potential for delayed diagnosis, unnecessary emergency presentations, inpatient admissions and duplication of service [22, 28, 40].

The management of acute CN is a specialised area of health and access to appropriate health services can be challenging. As previously identified, many health professionals lack an appropriate awareness of acute CN, and therefore appropriate health services tend to be centralised within metropolitan or large regional geographic regions. Accessing appropriate care by a competent health professional may not be possible in the regional or rural context [39]. Unfortunately, clinical guidelines do not consider the consequences of a lack of health service proximity or appropriately trained health professionals in a whole regional context [4]. Smaller health services require the means to provide appropriate assessment, diagnosis and management, and escalate health care or patients with acute CN appropriately. Appropriate access to services is a challenge in health, however in relation to acute CN, this becomes a greater challenge due to the need for timely diagnosis and implementation of treatment [4, 27]. Guidance documents and clinical pathways might assist with this, however, as identified in this review barriers to implementing them exist, especially in a geographically dispersed context [4, 6].

A further factor that can delay timely diagnosis and impact on management of acute CN is health professional knowledge of, and access to, the appropriate imaging. MRI is more appropriate for timely diagnosis than plain film x-ray, and nuclear medicine and bone scintigraphy might be useful [6, 16, 17, 30, 31, 40]. However, imaging guidelines are inconsistently applied across health professionals and health services [17, 20]. This review found that within health organisations the acute CN protocols for the use of MRI, nuclear medicine and bone scintigraphy are not always available, and geographical location and the proximity of the patient to appropriate health services might render the use of sophisticated imaging such as MRI, nuclear medicine and bone scintigraphy impossible [14, 39–41]. A similar barrier identified in this review is the availability of appropriately skilled health professionals to implement fundamental treatment plans such as initial TCC, and the subsequent recommencement to normal activity, which is often via well timed transition to removable non cast walkers and custom foot orthotics and shoes. The timelines for the use of advanced treatment modalities is dependant on the monitoring process and the skill of the health professional [14, 18, 22, 37], and can be dependent on health service location [6, 23, 24, 31].

Despite evidence-based guidelines existing for the assessment, diagnosis and management of acute CN the results of this review suggest that there are barriers to implementing this evidence-based care, which are likely to impact on widespread clinical translation. Whilst the dearth of existing high-level evidence may limit the certainty in some findings, the key themes that emerged possibly impact on this clinical implementation challenge. Further high-level research is required to better understand these factors. A translational research approach such as the Knowledge to Action (KTA) translational research framework would ensure that a depth of knowledge of evidence-based care across patients and health care professionals is better understood and incorporates consideration of important local contextual factors that may be influential [47].

The results of this review have shown that for acute CN it is particularly important to develop a better understanding of what patients and health professionals understand about key aspects of acute CN, and how health organisations across all communities are resourced to best support evidence-based care. This is a key concept of translational research and is considered “knowledge creation” in the KTA. This knowledge creation could then inform future research into initiatives to explore how knowledge and understanding may be improved, such as with targeted awareness campaigns and development of locally relevant health organisation policies. This “implementation” phase is the next step of the action cycle in the KTA framework [47]. This approach could assist evidence-based clinical pathway development that takes into consideration enablers and barriers, especially in the local context through consultation with stakeholders and review of patient medical information.

## Conclusion

Acute CN requires expedited assessment, diagnosis and management by appropriately trained health professionals in the appropriate setting to avoid serious morbidity. Patient and health professional awareness of acute CN and access to care are challenges to the delivery of evidence-based assessment, diagnosis and management of people with acute CN. Future research using a translational research approach underpinning is suggested to develop pragmatic and effective models of care for acute CN.

## Data Availability

All data generated and/or analysed are included in this published article.

## Abbreviations

CN: Charcot Neuroarthopathy
CROW: Charcot Restraint Orthotic Walker
CAM: Controlled Ankle Movement
CT: Computed Tomography
KTA: Knowledge to Action
MRI: Magnetic Resonance Imaging
PET: Positron Emission Tomography
TCC: Total Contact Cast
